# Altered blood glucose concentration is associated with risk of death among patients with community-acquired Gram-negative rod bacteremia

**DOI:** 10.1186/1471-2334-10-181

**Published:** 2010-06-22

**Authors:** Galo Peralta, M Blanca Sánchez, J Carlos Garrido, Begoña Ceballos, Fátima Mateos, Inés De Benito, M Pía Roiz

**Affiliations:** 1 Instituto de Investigación y Formación Marques de Valdecilla (IFIMAV), 5 Planta de la Escuela Universitaria de Enfermería, Avda de Valdecilla s/n, 39008, Santander, Spain; 2 Clinical Pharmacology Service, Hospital Universitario "Marqués de Valdecilla", Santander, Spain; 3 Biochemistry Service, Hospital Sierrallana, Torrelavega, Spain; 4 Emergency Service, Hospital Sierrallana, Torrelavega, Spain; 5 Microbiology Service, Hospital Sierrallana, Torrelavega, Spain; 6 Microbiology Service, Hospital Universitario Marques de Valdecilla, Santander, Spain

## Abstract

**Background:**

Altered blood glucose concentration is commonly observed in patients with sepsis, even among those without hypoglycemic treatments or history of diabetes mellitus. These alterations in blood glucose are potentially detrimental, although the precise relationship with outcome in patients with bacteremia has not been yet determined.

**Methods:**

A retrospective cohort study design for analyzing patients with Gram negative rod bacteremia was employed, with the main outcome measure being in-hospital mortality. Patients were stratified in quintiles accordingly deviation of the blood glucose concentration from a central value with lowest mortality. Cox proportional-hazards regression model was used for determining the relationship of same day of bacteremia blood glucose and death.

**Results:**

Of 869 patients identified 63 (7.4%) died. Same day of bacteremia blood glucose concentration had a U-shaped relationship with in-hospital mortality. The lowest mortality (2%) was detected in the range of blood glucose concentration from 150 to 160 mg/dL. Greater deviation of blood glucose concentration from the central value of this range (155 mg/dL, reference value) was directly associated with higher risk of death (p = 0.002, chi for trend). The low-risk group (quintile 1) had a mortality of 3.3%, intermediate-risk group (quintiles 2, 3 and 4) a mortality of 7.1%, and the high-risk group (quintile 5) a mortality of 12.05%. In a multivariable Cox regression model, the hazard ratio for death among patients in the intermediate-risk group as compared with that in the low risk group was 2.88 (95% confidence interval, 1.01 to 8.18; P = 0.048), and for the high risk group it was 4.26 (95% confidence interval, 1.41 to 12.94; P = 0.01).

**Conclusions:**

Same day of bacteremia blood glucose concentration is related with outcome of patients with Gram-negative rod bacteremia. Lowest mortality is detected in patients with blood glucose concentration in an interval of 150-160 mg/dL. Deviations from these values are associated with an increased risk of death.

## Background

Community acquired Gram-negative bacillus bacteremia (GNB) is a leading cause of hospitalization, sepsis and mortality [1]. Altered blood glucose concentration is frequently detected in patients with sepsis and has been associated with adverse outcome [2-9]. Variable cut-off values of hospital admission blood glucose concentrations with ranges from 100 to 200 mg/dL have been previously selected for defining outcome in observational studies with different populations as patients with stroke [10], head injuries [11], severe trauma [12], myocardial infarction [13], community acquired pneumonia [14], or patients admitted in a hospital through an emergency department [15].

Based on the negative effects of hyperglycemia in critically ill patients, detected in observational studies, several clinical trials that have tried to prove the benefits of its prevention with intensive insulin therapy with disappointing results. One of the reasons of these results is the potential effect of tight control of glucose blood concentrations causing hypoglycemia [16-18]. In fact hypoglycemia is also associated with negative effect over prognosis. In this situation there is a debate about the relevance of control blood glucose range in critically ill patients in general and specifically in patients with sepsis [19,20].

As there is limited information about outcome in relation with blood glucose concentrations in patients with sepsis and/or bacteremia, we conducted a hospital-based retrospective cohort study in order to investigate the association between same day of bacteremia blood glucose concentration (SDBGC) and mortality in patients with GNB.

## Methods

### Hospital setting and patients

The Sierrallana Hospital is an adult acute-care center in Torrelavega, a city in the North of Spain, which forms part of a health district of 160000 inhabitants. It is a community teaching hospital with 250 beds that has approximately 8000 admissions and 65000 patient visits to the emergency service annually. It includes most major departments and specialties, except transplantation, burns, thoracic surgery, cardiovascular surgery and neurosurgery units. Blood cultures are performed at the hospital in around 1800 patients per year.

A retrospective cohort study design was employed, with the main outcome measure being in-hospital mortality. Patients with Gram-negative rod bacteremia (GNB) from January 1997 to December 2006 were identified by using the microbiology laboratory database and their charts were reviewed retrospectively with a standardized data collection form. Only the community acquired episodes considered accordingly CDC criteria [21] were included in this study cohort. Only the first episode of bacteremia on each admission was included. One hundred and twenty six nosocomial episodes and 23 episodes with no blood glucose measurement recorded during the same day of the bacteremia were excluded. The following data were collected: age; sex; comorbid conditions; dates of hospital admission and discharge; presence of septic shock at the moment of blood culture extraction; specific antimicrobials administered during hospitalization; dates of start and end of antimicrobial administration; surgical procedures and hospitalization in an intensive care unit. The presence of each of the following comorbid conditions was assessed at the time of bacteremia: hepatic dysfunction, malignancy, diabetes mellitus, renal insufficiency, human immunodeficiency virus infection, neutropenia, corticosteroid use, previous transplantation, use of an immunosuppressive agent in the preceding 30 days. The study was approved by the Institutional Review Board of the hospital (Comité Ético de Investigación Clínica de Cantabria), which waived the need for informed consent due to the retrospective nature of the study.

### Definitions

A localized focus infection was considered to be the source or primary focus of bacteremia. Bacteremia was considered to have been nosocomially acquired if it appeared 48 h after admission and no evidence of infection was present on admission. The bacteremia was categorized as polymicrobial if additional microorganisms were recovered from the blood cultures. The source of the bacteraemia was determined on the basis of the isolation of the same microorganism from the presumed portal of entry and clinical evaluation. Renal insufficiency was indicated by a creatinine value of 2.0 mg/dL. Neutropenia was defined as an absolute neutrophil count ≤500 cells/mm^3 ^at the onset of the bacteraemia. Immunosuppression was defined as the presence of neutropenia or HIV infection (with CD4 count ≤350 cells/mm3), or receipt of immunosuppressive agents. Comorbidities were assessed by using the Charlson comorbidity score [22]. Septic shock was defined as proposed by Bone et al [23]. Empirical antimicrobial therapy was judged to be either adequate or inadequate on the basis of the in vitro susceptibility of an isolated organism, and/or the initiation of antibiotic treatment within 24 h of blood culture extraction. Oxyimino-b-lactams (cefuroxime, cefotaxime, ceftriaxone, ceftazidime and aztreonam) were considered to be inappropriate regardless of the MIC for the treatment of infections caused by ESBL-producing Gram-negative microorganisms. Therapy with urinary antiseptics such as norfloxacin, fosfomycin, pipemidic acid or nalidixic acid was considered inadequate [24].

### Blood cultures

The common practice for blood cultures at our hospital is to obtain 20 mL of venous blood and to inoculate it in equal parts into one aerobic (BacT/ALERT FA aerobic, bioMérieux Corporation, Durham, North Carolina) and one anaerobic blood culture bottle (BacT/ALERT FN, bioMérieux). Blood drawn is performed by nurses from a peripheral vein three times at intervals of 30 minutes. As a routine practice at our hospital a positive finding of microbial growth in the blood culture is reported to the attending physicians before the results of antimicrobial susceptibility test are known and organism identification is established.

### Same day of bacteremia blood glucose measurement

In patients with more than one blood glucose measurement obtained on the same day of the bacteremia only the first one was considered for the analysis. Blood glucose tests were performed at the clinical chemistry laboratory on venous samples by the glucose oxidase method.

### Statistical analysis

Bivariable analyses were conducted to determine the association between potential risk factors and mortality. Of primary interest was the association between blood glucose concentration and in hospital mortality. Categorical data were compared by the chi-square or Fisher's exact tests. Quantitative data were compared by Student's *t *test or the Mann-Whitney *U *test, as appropriate. To evaluate the effect of different levels of blood glucose concentration on mortality, relative risks and 95 percent confidence intervals were calculated as hazard ratios derived from the Cox proportional-hazards regression model. Multivariable models were fitted with use variables with a *P *value < 0.1 in the univariate. The level of significance was set at P < 0.05. The SPSS (version 14) software package was used for all analyses.

## Results

### Patient population

During the study period, blood cultures were performed in 18094 patients and were positive in 2678. Among these, 1018 patients were identified as having GNB. After considering inclusion/exclusion criteria, 869 cases were analyzed. The demographic and clinical characteristics of these patients are listed in Table 1. The median age of the patients was 75 years (interquartile range, 64-82 years). In 721 (83%) blood cultures were obtained at the emergency department, and in 821 (94.5%) blood glucose determination was performed in samples obtained in the emergency department. Of the selected patients, 672 (77.3%) were hospitalized and the remainder were diagnosed of bacteremia after the patients had been discharged from the emergency department and re-evaluated in an outpatient clinic.

**Table 1:** Demographic and clinical characteristics of patients with bloodstream infections due to Gram-negative bacilli.

Characteristics	Value
**Demographics**	

Male sex	458 (52.7)

Age >65 y	635 (73.1)

**Comorbidity**	

Diabetes mellitus	139 (16)

Neoplasm	81 (9.3)

COPD	81 (9.4)

Dementia	71 (8.2)

Liver cirrhosis	48 (5.5)

Stroke	51 (5.9)

Immunosuppression	37 (4.3)

Chronic renal failure	29 (3.4)

Charlson index ≥3	145 (16.7)

**Microorganism**	

*E. coli *	710 (81.7)

*Klebsiella spp.*	53 (6.1)

*Proteus spp.*	44 (5.1)

*Salmonella spp.*	39 (4.5)

*Enterobacter spp.*	22 (2.5)

Polymicrobial bacteremia	67 (7.7)

**Adequate empirical treatment**	648 (74.6)

**Presentation**	

Septic shock	9 (1.0)

**Outcome**	

Required ICU	29 (3.3)

In hospital mortality	63 (7.2)

Data are no. (%) of patients. ICU: Intensive care unit.

The main sites of infection were as follows: urinary in 484 patients (55.7%), biliary in 157 (18.1%); unknown in 116 (13.3%); abdominal in 37 (4.3%); enteric in 28 (3.2%); pneumonia in 16 (1.8%), skin in 13 (1.5%) and spontaneous bacterial peritonitis in 8 (0.9%).

### Blood glucose concentrations

**Figure 1:**
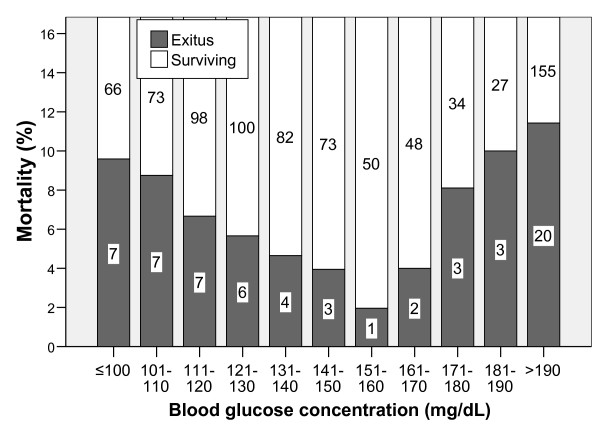
**In hospital mortality according to blood glucose concentration**. The numbers inside the bars reflect the number of patients in each group.

The range of SDBGC was 52 to 582 mg/dL. The relationship of mortality and blood glucose concentration showed an asymmetric U shape distribution with the lowest mortality among patients with glucose blood concentration of 151-160 mg/dL (Figure 1).

**Figure 2:**
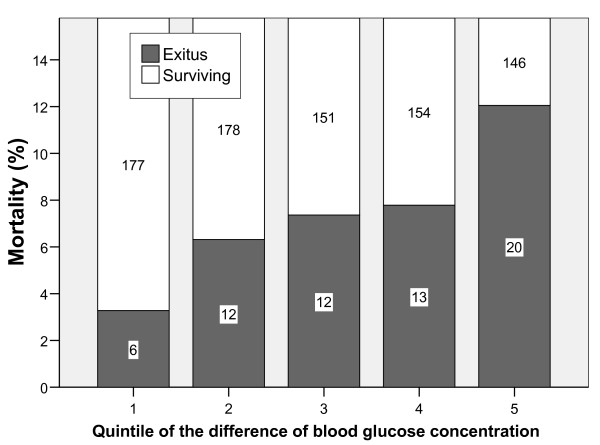
**Mortality according to deviation of blood glucose concentration with the reference blood glucose concentration of 155 mg/dL which is the central value of the interval with the lowest mortality**. Patients are distributed in quintiles of the difference in blood glucose concentration. The numbers inside the bars reflect the number of patients in each group (p = 0.002 chi square for linear trend).

Patients were divided into subgroups according the difference of the blood glucose concentration with the central value of the interval with lowest mortality (155 mg/dL, reference value). Characteristics of these subgroups (quintiles) are reflected in table 2. The proportion of patients with diabetes mellitus or liver cirrhosis was much higher among those classified in the 5^th ^quintile. A relationship between the absolute difference of blood glucose concentration respect the reference value and mortality was detected (Figure 2).

**Table 2:** Baseline characteristics of patients with GNB according to quintiles of the absolute difference of the SDBGC respect the reference value of lowest mortality (155 mg/dL).

	**1**^**st **^**Quintile** n = 183	**2**^**nd **^**Quintile** n = 190	**3**^**rd **^**Quintile** n = 163	**4**^**th **^**Quintile** n = 167	**5**^**th **^**Quintile** n = 166	p
**Range of difference in BGC with the reference value (mg/dL)**	≤15	16-29	30-42	43-62	≥63	-

**Demographics**						

Male sex	59.0	53.7	54.0	54.5	41.6	0.02

Age >65 y	74.9	76.3	75.5	61.4	77.1	0.006

**Comorbidity**						

Diabetes mellitus	10.4	11.1	6.8	4.2	49.1	<0.001

Neoplasm	10.4	10.5	9.3	10.2	6.1	0.59

COPD	9.3	13.2	9.9	7.8	6.1	0.2

Dementia	6.0	6.8	8.0	7.9	12.7	0.19

Liver cirrhosis	4.4	3.2	6.2	4.2	10.3	0.04

Stroke	5.5	6.3	3.1	6.6	7.9	0.44

Immunosuppression	4.4	2.1	3.7	6.0	5.5	0.39

Chronic renal failure	2.7	2.1	4.3	2.4	5.5	0.37

Charlson ≥3	11.5	16.1	13.8	18.5	14.8	0.57

**Origin of infection**						

Urinary	49.5	55.0	55.3	63.0	59.1	0.12

Biliary	20.3	21.2	22.4	13.9	12.8	0.07

Unknown	17.6	12.2	12.4	9.1	15.9	0.16

Intra-abdominal	4.4	3.7	4.3	4.8	4.3	1

Pneumonia	1.1	1.6	1.2	2.4	3.0	0.64

**Microorganism**						

*E. coli *	82.0	83.2	82.2	82.6	78.3	0.8

*Klebsiella spp.*	6.0	3.7	6.7	6.6	7.8	0.56

*Proteus spp.*	4.4	5.8	6.1	4.8	4.2	0.9

*Salmonella spp.*	5.5	3.7	3.7	4.2	5.4	0.86

*Enterobacter spp.*	2.2	3.7	1.2	1.2	4.2	0.25

**Adequate empirical treatment**	82.6	80.3	85.8	81.8	83.7	0.7

Polymicrobial bacteremia	8.2	7.4	9.2	5.4	8.4	0.74

**Presentation**						

Septic shock	0	0.5	1.8	1.2	1.8	0.35

**Outcome**						

Required ICU	4.4	1.6	3.1	4.2	3.6	0.58

Death	3.3	6.3	7.4	7.8	12.0	0.03

Data are % of patients. ICU: Intensive care unit.

On the basis of the findings presented in figure 2, we combined subgroups into three categories designated as low-risk (quintile 1, corresponding to blood glucose concentration from 140 to 170 mg/dL), intermediate-risk (quintiles 2, 3 and 4, corresponding to blood glucose concentration from 93 mg/dL to 139 mg/dL and from 171 mg/dL to 217 mg/dL), and high-risk (quintile 5, corresponding to blood glucose concentration <93 mg/dL or greater than 217 mg/dL). Mortality in the intermediate-risk category was 7.1% (95% Confidence interval: 5% to 9.3%), which was similar to the average mortality of 7.4% for the entire cohort.

### Predictors of mortality

**Figure 3:**
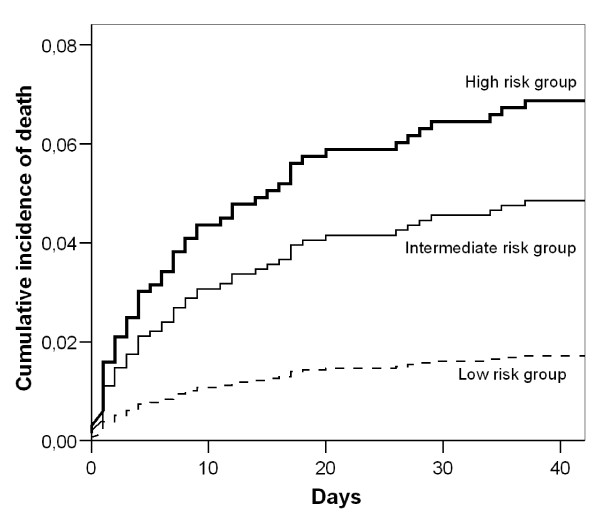
**Adjusted estimates of overall survival in patients with GNB according to risk groups**. The three risk groups are based in the quintiles of the absolute difference in glucose blood concentration with the central value of the interval with lowest mortality: low risk group (quintile 1), intermediate risk group (quintiles 2, 3 and 4), high risk group (quintile 5). Survival estimates have been adjusted for the presence of age >65 years, Charlson index ≥3, presence of immunosuppression, liver cirrhosis, urinary origin, lung origin, unknown origin, *E. coli *bacteremia, polymicrobial bacteremia, presence of shock and adequacy of empirical antibiotic treatment. P = 0.036 by the log-rank test for the overall comparison between groups.

Risk factors for death in a univariate analysis are reflected in table 3. Kaplan-Meier estimates of survival for all subjects according to blood glucose concentration risk categories are shown in figure 3. In a multivariable Cox regression model, the hazard ratio for death among patients in the intermediate-risk group according to blood glucose concentration as compared with that in the low risk group was 2.88 (95 percent confidence interval, 1.01 to 8.18; P = 0.048), and for the high risk group it was 4.26 (95 percent confidence interval, 1.41 to 12.94; P = 0.01). Other factors associated with death are reflected in table 4. When we selected non-diabetic patients for the analysis the hazard ratio for death among patients in the intermediate-risk group according to blood glucose concentration as compared with that in the low risk group was 2.76 (95 percent confidence interval, 0.96 to 7.98; P = 0.06), and for the high risk group 5.11 (95 percent confidence interval, 1.55 to 16.92; P = 0.008).

**Table 3:** Univariate analysis of factors associated with mortality in patients with GNB.

	Surviving N = 806	Death N = 63	RR (95%CI)	P
**Demographics**				

Male	426 (52.9)	32 (50.8)	0.93 (0.58-1.49)	0.43

Age >65 y	581 (72.2)	54 (85.7)	2.20 (1.11-4.39)	0.01

**Comorbidity**				

Diabetes mellitus	130 (16.2)	9 (14.3)	0.87 (0.56-2.41)	0.43

Neoplasm	74 (9.2)	7 (11.1)	1.2 (0.57-2.57)	0.38

Immunosuppression	31 (3.9)	6 (9.5)	2.36 (1.08-5.11)	0.046

Liver cirrhosis	41 (5.1)	7 (11.1)	2.13 (1.02-4.41)	0.052

Chronic renal failure	45 (5.6)	6 (9.5)	1.68 (0.76-3.71)	0.16

Charlson index ≥3	129 (16)	21 (33.3)	2.4 (1.46-3.92)	0.001

**Microorganism**				

*Escherichia coli *	664 (82.4)	46 (73)	0.61 (0.36-1.03)	0.05

*Klebsiella spp.*	48 (6.0)	5 (7.9)	1.33(0.56-3.17)	0.34

*Proteus spp.*	40 (5.0)	4 (6.3)	1.27 (0.48-3.34)	0.4

*Salmonella spp.*	34 (4.2)	5 (7.9)	1.84 (0.78-4.32)	0.14

*Enterobacter spp.*	19 (2.4)	3 (4.8)	1.93 (0.65-5.67)	0.21

*Polymicrobial bacteremia*	56 (6.9)	11 (17.5)	2.56 (1.4-4.64)	0.006

**Adequate empirical treatment**	610 (83.7)	38 (70.4)	0.5 (0.28-0.86)	0.014

**Origin of infection**				

Urinary	466 (58.3)	18 (29.0)	0.32 (0.19-0.54)	<0.0001

Biliary	147 (18.4)	10 (16.1)	0.86 (0.45-1.66)	0.4

Unknown	101 (12.6)	15 (24.2)	2.05 (1.19-3.54)	0.013

Intra-abdominal	32 (4.09	5 (8.1)	1.95 (0.83-4.58)	0.12

Pneumonia	10 (1.3)	6 (9.7)	5.66 (2.86-11.18)	<0.001

**Presentation**				

Septic Shock	4 (0.5)	5 (7.9)	8.24 (4.37-15.54)	<0.0001

Data are no. (%) of patients. RR: Relative risk

**Table 4:** Results of Cox analyses examining risk factors for mortality associated with bacteraemia due to Gram-negative bacilli.

Mortality risk factor	HR (95% CI)	p value
Charlson index ≥3	2.04 (1.12-3.69)	0.02

Urinary origin	0.44 (0.23-0.82)	0.01

Lung origin	4.14 (1.54-11.08)	0.005

Polymicrobial bacteremia	2.6 (1.28-5.28)	0.008

Adequate empirical treatment	0.43 (0.23-0.79)	0.006

Shock	9.31 (3.42-25.38)	<0.001

Blood glucose category (high vs low risk)	4.26 (1.4-12.9)	0.01

HR: hazard ratio

## Discussion

In this cohort study of patients with GNB, SDBGC was independently associated with prognosis. SDBGC had a U-shaped relationship with in-hospital mortality with the lowest mortality in patients with SDBGC of 150-160 mg/dL. Based on the deviation of blood glucose concentration from the value with the lowest mortality, we classified patients in groups with low, intermediate, and high risk categories of death. As compared with the low risk blood glucose concentration category, intermediate risk concentration category and high risk category were associated with hazard ratios for death of 2.88 and 4.6, respectively. The increased risk was similar for patients without diabetes mellitus. Although several other studies have made risk assessments for patients with GNB, this is, to our knowledge, the first observational study to evaluate the effect of the deviation from a reference level of blood glucose concentration on outcome in this population.

Hyperglycemia associated with insulin resistance is common in critically ill patients and, although it can be considered an adaptive response, it has been associated with outcome [2,7,16-18]. Several observational studies have demonstrated that elevated initial blood glucose is independently associated with prognostic of severe acute illness [25]. However, the specific consequences of hyperglycemia in patients with sepsis have not quite been addressed [7,8,19,26]. Hypoglycemia is also a manifestation of sepsis [2-5] and is associated with a worse outcome in hospitalized patients with acute illnesses [4,6] and specifically in patients with *E. coli *bacteremia [5].

As the initial altered blood glucose has been considered detrimental in the last years several studies have evaluated the effect of controlling blood glucose concentrations in critically ill patients with insulin therapy over mortality. A prospective randomized non-blind trial showed that critical medical intensive care unit patients had a slight and not significant reduction in ICU mortality of 2.8% when treated with intensive insulin therapy to achieve target levels of blood glucose concentration from 80 to 100 mg/dL when compared with patients receiving conventional glucose control (target blood glucose level 180-220 mg/dL) [17]. The study only demonstrated a beneficial effect after the first three days of the intensive insulin therapy [17]. Another multicenter randomized prospective trial of intensive versus conventional glycemic control in severe sepsis was closed prematurely because of a nearly six fold increase in severe hypoglycemia among the patients in the intensive glycemic control treated group [26]. Moreover, the selection of a strict target blood glucose concentration is not supported by some observational studies, which reveal that optimal blood glucose concentration for these critically ill patients may be higher [25]. On the other hand intensive insulin therapy is potentially detrimental per se [16,17,27]. Although hypoglycemia in hospitalized patients is harmful [4,6,26,27], no information is reflected in the literature about the optimal management of hypoglycemia in patients with sepsis, except for causal treatment when feasible.

Our results are consistent with some previous studies. Finney et al [25] found in an observational study that a target blood glucose level of around 145 mg/dL may be associated with a better outcome in critically ill patients. We have detected similar differences in mortality of 2.7% than a previous randomized clinical trial, when we compare patients with blood glucose concentration of 80-100 mg/dL and 180-200 mg/dL that were the end points defined in that trial [17]. However, the optimal blood glucose levels may be different after the first days of hospital admission [17] and our data are referred to the day of the bacteremia. One recent observational study from Australia in critically ill patients has detected also a U shape relationship of blood glucose concentration with mortality, with lowest levels of mortality in patients with blood glucose concentrations among 100-150 mg/dL [28]. Similarly with this study the U shape relationship of blood glucose concentration with mortality that we have detected is asymmetric and there was more increase in mortality in patients with high than with low blood glucose with respect the range with lowest mortality. If sepsis related mortality has a direct relationship with high or low blood glucose concentration, it can be speculated that increasing blood glucose concentration to achieve an optimal target level could be of potential benefit. The threshold to indicate correction of low blood glucose concentration should first be determined [28].

Our study has several limitations. Data are retrospective and the results came from a population with specific characteristics: patients with GNB, a low proportion of immunosuppression, stay in ICU, septic shock or death, so the extrapolation of these results must be made with caution. As has been commented, the measurements of blood glucose concentration were made at the beginning of the illness, so it remains unknown whether the blood glucose concentrations associated with the lowest mortality would be the same after the initial phases of GNB. However as we select initial blood glucose concentration for the analysis, those are not influenced by therapies as insulin, glucose-containing solutions or parenteral nutrition used during hospitalizations.

In summary, our study indicates that low and high blood glucose concentrations are associated with higher mortality of patients with GNR bacteremia regardless of the presence of diabetes mellitus. Studies to determine the precise correlation between blood glucose concentration and mortality with suspected bacteremia are needed.

## Conclusions

Same day of bacteremia blood glucose concentration is related with outcome of patients with Gram-negative rod bacteremia. Lowest mortality is detected in patients with blood glucose concentration in an interval of 150-160 mg/dL. Deviations from these values are associated with an increased risk of death.

## Competing interests

Galo Peralta has been a consultant in the past for Janssen-Cilag, Wyeth, Bristol-Myers Squibb, and Boehringer Ingelheim, and has also served as a speaker for Wyeth and GlaxoSmithKline.

## Authors' contributions

GP, MPR, MBS, and JCG were involved in the study conception, BC, FT and IDB were involved in the coordination, and data acquisition, GP performed the data analyses, all authors were involved in the interpretation and validation of the results, GP, MPR and MBS were involved in the drafting of the manuscript and all authors read and approved the final manuscript.

## Pre-publication history

The pre-publication history for this paper can be accessed here:

http://www.biomedcentral.com/1471-2334/10/181/prepub
